# *Streptomyces cameroonensis* sp. nov., a Geldanamycin Producer That Promotes *Theobroma cacao* Growth

**DOI:** 10.1264/jsme2.ME16095

**Published:** 2017-03-04

**Authors:** Thaddée Boudjeko, Romaric Armel Mouafo Tchinda, Mina Zitouni, Joëlle Aimée Vera Tchatchou Nana, Sylvain Lerat, Carole Beaulieu

**Affiliations:** 1 Laboratory of Phytoprotection and Valorization of Plants Resources, Biotechnology Centre—NkolbissonP.O. BOX 3851, Messa, YaoundéCameroon; 2 Department of Biochemistry, Faculty of Science, University of Yaoundé IP.O. Box 812 YaoundéCameroon; 3 Centre SÈVE, Département de Biologie, Université de SherbrookeSherbrooke, Quebec J1K 2R1Canada

**Keywords:** geldanamycin, PGPR, *Streptomyces violaceusniger* clade, *Theobroma cacao*

## Abstract

The taxonomy of an actinobacterial strain, designated JJY4^T^, was established using a polyphasic approach. JJY4^T^ was isolated from the rhizosphere of *Chromolaena odorata* in Yaoundé (Cameroon) during a project for the selection of biological control agents. Strain JJY4^T^ exhibited antimicrobial activities against bacteria, fungi, and oomycetes. Strain JJY4^T^ also exhibited the traits of plant growth-promoting rhizobacteria such as the solubilization of inorganic phosphate, production of siderophores and indole-3-acetic acid, and 1-aminocyclopropane-1-carboxylate deaminase activity. *In planta* assays performed on cocoa plantlets confirmed that strain JJY4^T^ exhibited strong abilities to promote plant growth and protect against *Phytophthora megakarya*, the main causal agent of cocoa pod rot. The formation of rugose-ornamented spores in spiral spore chains by strain JJY4^T^ is a typical feature of members found in the *Streptomyces violaceusniger* clade and, similar to some members of the clade, strain JJY4^T^ produces geldanamycin. A phylogenetic analysis based on 16S rRNA gene sequences confirmed this classification and suggests that strain JJY4^T^ be added to the subclade constituted of the type strains *Streptomyces malaysiensis* DSM 41697^T^ and *Streptomyces samsunensis* DSM 42010^T^. However, DNA–DNA relatedness and physiological characteristics allowed for the differentiation of strain JJY4^T^ from its closest phylogenetic relatives. Based on these results, strain JJY4^T^ (=NRRL B-65369, =NBRC 112705) appears to represent a novel species in the *S. violaceusniger* clade for which the proposed name is *Streptomyces cameroonensis* sp. nov.

Actinobacteria are Gram-positive, aerobic bacteria that are characterized by a genome with a high G+C content. *Streptomyces* is the largest genus of the order *Actinomycetales*. *Streptomyces* species are multicellular bacteria and their cells contain multiple copies of a linear chromosome. Members of this genus exhibit complex morphological differentiation starting with the germination of a spore that grows to form a vegetative mycelium (5). This mycelium may give rise, under adverse conditions, to an aerial mycelium that will eventually fragment into spores. Streptomycetes are abundant in soil, in which they play important roles in nutrient cycling. The genus *Streptomyces* is known for its ability to produce bioactive metabolites and lytic enzymes (5, 8), and has, thus, been extensively investigated for the selection of biocontrol agents of plant diseases and plant growth-promoting microbes (8, 9).

Actinobacteria are saprophytic or symbiotic microorganisms that are widely associated with plants (28). Although actinobacteria displaying the characteristics of biocontrol agents and plant growth-promoting bacteria have been successfully isolated from epigeous plant tissues (26), the rhizosphere represents the main reservoir for these microorganisms (9). A large number of studies have reported the isolation of actinobacteria depicted as potential biocontrol agents in the literature.

During a project aimed at isolating rhizospheric actinobacteria as candidates for the biocontrol of cocoa (*Theobroma cacao*) pod rot, rhizosphere-associated soil samples were collected from plants growing in an uncropped field in Cameroon. Among the 60 actinobacterial isolates obtained, one strain, namely JJY4^T^, emerged as a broad-spectrum and efficient antimicrobial agent. Phylogenetic and phenotypic tests performed to identify JJY4^T^ strongly suggest that this strain be assigned to a novel species in the *Streptomyces violaceusniger* clade. We proposed to name this novel species *Streptomyces cameroonensis*, with strain JJY4^T^ as the type strain.

## Materials and Methods

### Strain isolation

Rhizosphere-associated soil was sampled from a fallow in Nkolbisson (Yaoundé, Cameroon, coordinates 3°52′24.4″N-11°26′7.8″E). The roots of *Chromolaena odorata* were extricated, and 100 g of soil that was firmly attached to the roots was collected with a sterile spatula and placed into sterile plastic bags. Samples were diluted in sterile water, incubated at 50°C for 10 min, and plated on yeast extract-malt extract (ISP-2) agar (35) supplemented with nalidixic acid (10 μg mL^−1^). Plates were incubated at 30°C for 21 d. Actinobacteria-like colonies were selected and purified by serial subcultures. Sixty isolates were collected. Spores were harvested using glass beads rolled over sporulating colonies. Beads were then washed with 20% (v/v) glycerol and spore suspensions were kept at −20°C. The isolates were then screened for their ability to inhibit the growth of *Phytophthora megakarya* (see below), a plant pathogenic agent responsible for the black pod rot of cocoa. The strain that showed the highest antagonistic activities against *P. megakarya*, named JJY4^T^, was selected for further characterization.

### Screening for antimicrobial activity

Tests for antimicrobial activities against fungi and oomycetes were performed on potato dextrose agar (PDA) plates at 28°C for 5 d. The antagonistic activities of JJY4^T^ against *Aspergillus niger* UdS-203, *Botrytis cinerea* B191, *Fusarium oxysporum* UdS-212, *Phytophthora erythroseptica* UdS-305, *Phytophthora infestans* UdS-302, *P. megakarya* Ngo13, *Pythium aphanidermatum* UdS-321, and *Pythium myriotylum* P60 were evaluated according to Aouar *et al.* (4). Briefly, JJY4^T^ was streaked across the center of PDA plates and incubated at 25°C for 5 d. Two discs (7 mm in diameter) from an 8-d-old fungal culture were placed 1.5 cm from the edge of the plates. After being incubated at 28°C for 5 d, fungal growth was assessed by measuring mycelial development. Control treatments were prepared similarly without JJY4^T^.

The capacity of JJY4^T^ to inhibit the *in vitro* growth of *Agrobacterium tumefaciens* CB-1 and *Streptomyces scabiei* EF-35 was evaluated as follows. JJY4^T^ was grown on ISP-2 agar plates at 28°C. After a 10-d incubation, agar plugs (5 mm in diameter) were flipped over the center of plates on which tested bacteria had been previously plated. After a 24–48-h incubation at 25°C or 37°C, the presence or absence of a growth inhibition zone around the JJY4^T^-inoculated agar plug was scored.

### Identification of bioactive molecules produced by JJY4^T^

One L (2×500 mL) of ISP-2 medium was inoculated with spores from strain JJY4^T^ and incubated at 30°C for 5 d with shaking (250 rpm). Bacteria were then pelleted by centrifugation at 3,500×*g* for 10 min and secondary metabolites were extracted from the supernatant with ethyl acetate. This organic fraction was evaporated and the resulting material was dissolved in methanol and separated by thin layer chromatography (TLC) silica gel 60 F_254_ (1000 μm), using chloroform:methanol (95:5) as migration solvents. An antibiogram performed by pouring *S. scabiei* in soft tryptic soy agar (0.4%) on the TLC plate revealed antibiotic activity at an *Rf* of *c.* 0.50, at which the mainly represented secondary metabolite was detected.

A larger quantity of secondary metabolites was then obtained from a 2-L culture. The candidate molecules of interest (*Rf* of *c.* 0.50) were scraped after migrating on TLC plates and extracted from silica gel using a chloroform:methanol (70:30) mixture. After drying, the molecules were dissolved in acetonitrile and individually separated by high performance liquid chromatography equipped with a fraction collector (Agilent 1260 Series, Agilent Technologies), using a Zorbax SB-C18 column (Agilent Technologies) and applying a 55–85% acetonitrile gradient for 10 min at a flow rate of 1 mL min^−1^ (6). Separation was based on the presence of peaks at 306 nm.

The exact mass of the molecule present in the fraction that exhibited antimicrobial activity was then assessed by inductively coupled plasma mass spectrometry (XSeries II, Thermo Scientific). Its chemical structure was revealed by crystallography. Crystals were obtained by the slow diffusion of EtOH in CH_2_Cl_2_ solution followed by slow evaporation, and analyzed by X-ray diffraction using an Apex DUO system (Bruker Co.) equipped with a Cu Kα ImuS micro-focus source with MX optics (λ=1.54186 Å).

### Analysis of plant growth-promoting rhizobacterial properties

Strain JJY4^T^ was examined for the presence of several traits commonly found in plant growth-promoting rhizobacteria (PGPR). The capacity to solubilize phosphate was tested according to the procedure described previously using Pikovskaya medium (14). The development of clear zones around the colonies caused by the utilization of tricalcium phosphate, revealing phosphate solubilization, was noted.

Siderophore production was measured on blue agar CAS medium containing chrome azurol S (CAS) and hexadecyltrimethylammonium bromide as indicators (33). The production of siderophore was indicated by a change in color from blueish to yellowish orange.

ACC (1-aminocyclopropane-1-carboxylate) deaminase activity was assessed in growing JJY4^T^ on Dworkin and Foster (DF) minimal salts medium containing 2 g L^−1^ of ACC as the nitrogen source. The presence of 2-ketobutyrate generated by the hydrolysis of ACC was measured at 540 nm (29).

The biosynthesis of indole-3-acetic acid (IAA) was assessed in minimal medium supplemented with l-tryptophan. A bacterial inoculum (100 μL of a 48-h pre-culture in ISP-2 medium) was added to 25 mL of minimal medium supplemented with 0.5% starch and containing 6 mM of l-tryptophan, and flasks (*n*=3) were incubated at 30°C for 6 d with shaking (250 rpm). Cultures were centrifuged for 5 min to pellet bacteria and IAA was extracted from supernatants with ethyl acetate as described previously (25).

### Bacterial effects on plant development and protection against *P. megakarya*

Cocoa seeds were obtained from cocoa pods washed with fine sand and rinsed with water. Seeds were placed in pots containing a sterile mixture of soil and sand (1/3 and 2/3, respectively). The pot substrate was treated as follows: not inoculated, inoculated with spores (10^8^ kg^−1^ of soil) of JJY4^T^, inoculated with zoospores (10^7^ kg^−1^ of soil) of *P. megakarya*, or co-inoculated with JJY4^T^ and *P. megakarya*. The germination success of 12 seeds in each of the four treatments was scored 10 and 30 d after seeding.

In order to assess growth performance, cocoa seedlings were grown in pots containing the same sterilized substrate used for the germination experiment (see above). The growth substrate was inoculated or not with JJY4^T^ and/or *P. megakarya* as described above. The pots were kept outside at the Nkolbisson Biotechnology Centre for 3 months under protective covering. Plants were watered first on a daily basis for 3 weeks, and thereafter every other d. Plants were then destructively harvested to assess root and aerial dry biomasses. The experiment was performed in five replicates.

Cocoa plantlets that had been grown in the presence or absence of JJY4^T^ for 3 months as previously described were compared for their resistance to *P. megakarya* using a foliar disc method (27). Eight 15-mm discs (15 mm) from the leaves of a similar developmental stage were challenged with 10 μL of a *P. megakarya* spore suspension (10^6^ zoospores mL^−1^). Discs were placed in Petri dishes and then inoculated and incubated in the dark at 26°C for 6 d. The severity of necrosis was noted according to a scale ranging from 0 (no necrosis) to 5 (extreme necrosis) (27). The experiment was conducted in triplicate.

### Cultural and morphological characteristics

The cultural characteristics of strain JJY4^T^ were assessed by inoculating on various International *Streptomyces* Project (ISP) (ISP-2, ISP-3, ISP-4, ISP-5 and ISP-7; 35) and non-ISP (glycerol bouillon [GB; 36] and modified Bennett’s [16]) media. These cultures were examined for pigmentation and spore mass. Color was visually estimated by comparing the culture with chips from ISCC-NBS color charts (18) and melanin pigment production was evaluated based on growth on tyrosine (ISP-7) agar. The morphology of spore-bearing hyphae was assessed after 10–12 d of growth on starch-inorganic salt (ISP-4) agar using the cover-slip method (42) and by scanning electron microscopy as previously described (4).

### Biochemical and physiological characteristics

Strain JJY4^T^, *S. samsunensis* DSM 42010^T^, and *S. malaysiensis* DSM 41697^T^ were grown and tested simultaneously in order to examine the following characteristics: degradation and enzymatic activities, carbon and nitrogen source utilization, growth in the presence of inhibitors, growth at different pH and temperatures, and tolerance to antibiotics. The degradation tests of tyrosine (0.4%), hypoxanthine (0.4%), and casein (1% skimmed milk) were performed according to Williams *et al.* (42) on modified Bennett’s agar as basal medium. Activities were detected after 7, 14, and 21 d (the clearing of insoluble compounds around colonies was scored as positive). The degradation of arbutin and aesculin (0.1%, w/v) was assessed by the method described previously (42). Starch (1%) and gelatin (0.4%) degradation was evaluated on modified Bennett’s agar after 7 d by flooding plates with iodine and MgCl_2_ solutions, respectively, and scoring the zones of clearing as positive. The degradation of Tween 80 (1%) was assessed using Sierra medium, which was examined for opacity after 3, 7, and 14 d.

Carbon sources were added at a final concentration of 1% (w/v) to ISP-9 basal medium, except for organic acids, which were used at a concentration of 0.1% (w/v). The assimilation of nitrogen sources (added at 0.1% [w/v]) was also investigated (42). Growth at 30°C was scored after 14 d by comparing test plates with negative and positive controls.

Cellulolytic, pectinolytic, chitinolytic, and chitosanolytic activities were detected by the appearance of clear zones around colonies grown on ISP-9 medium supplemented (1%) with cellulose, pectin, chitin, and chitosan, respectively.

Tolerance to temperature and pH and resistance to chemical inhibitors were tested on modified Bennett’s agar (42). Growth at pH 4.0, pH 5.0, pH 7.0, and pH 9.0 (at a fixed temperature of 25°C) as well as 4, 25, 37 and 45°C (at fixed pH 7.0) was measured after 14 d or 6 weeks. In growth inhibition tests, modified Bennett’s agar was supplemented with one of the following potential inhibitors: phenol (0.1% [w/v]), sodium azide (0.01% [w/v]), crystal violet (0.0001% [w/v]), thallium acetate (0.01% [w/v]), potassium tellurite (0.01% [w/v]), cadmium chloride (0.01% [w/v]), and NaCl (4, 8, 10, and 13% [w/v]). Tolerance to antibiotics was assessed using modified Bennett’s agar. In these tests, the presence or absence of growth was scored after 7 and 14 d.

### Chemotaxonomic characterization

JJY4^T^ was grown at 30°C to the log phase in ISP-2 medium. Mycelia were collected by centrifugation (3,500×*g* for 10 min) and rinsed thrice with distilled water. Analyses of 2,6-diaminopimelic acid (30), respiratory quinones (37, 38), polar lipids (39), and fatty acids (17) were performed on lyophilized cells by the Identification Service, DSMZ (Braunschweig, Germany).

### Phylogenetic characterization

The genomic DNA of JJY4^T^, *Streptomyces malaysiensis* DSM 41697^T^, and *Streptomyces samsunensis* DSM 42010^T^ was extracted using the salting-out procedure (20). The almost complete 16S rRNA gene of JJY4^T^ was amplified using the universal primers BSF-8/20 (5′-AGAGTTTGATCCTGGCTCAG-3′) and BSR-1541/20 (5′-AAGGAGGTGATCCAGCCGCA-3′) and then sequenced. Sanger-type sequencing was performed at the “Plateforme de séquençage et de génotypage des génomes” (Quebec City, QC, Canada) using an ABI 3130*xl* Genetic Analyzer (Applied Biosystems). The 16S rRNA gene sequence obtained for strain JJY4^T^ was deposited in GenBank with the accession number KC329480.

Phylogenetic trees were constructed with the MEGA7 software package (22) using the neighbor joining (31) and maximum likelihood methods (10) with the Kimura 2 parameter model (21). A bootstrap analysis was performed using 1,000 resamplings. The root position of the neighbor-joining tree was deduced using *Actinomadura nitritigenes* as an outgroup.

In order to distinguish strain JJY4^T^ from its two phylogenetically closest neighbors, *S. malaysiensis* DSM 41697^T^ and *S. samsunensis* DSM 42010^T^, the levels of DNA–DNA relatedness were examined (11) based on the thermal denaturation midpoints of homologous and heterologous DNA preparations (Δ*Tm*). The DNA G+C content of strain JJY4^T^ was calculated according to the method developed by Gonzalez and Saiz-Jimenez (11).

## Results and Discussion

**Fig. 1:**
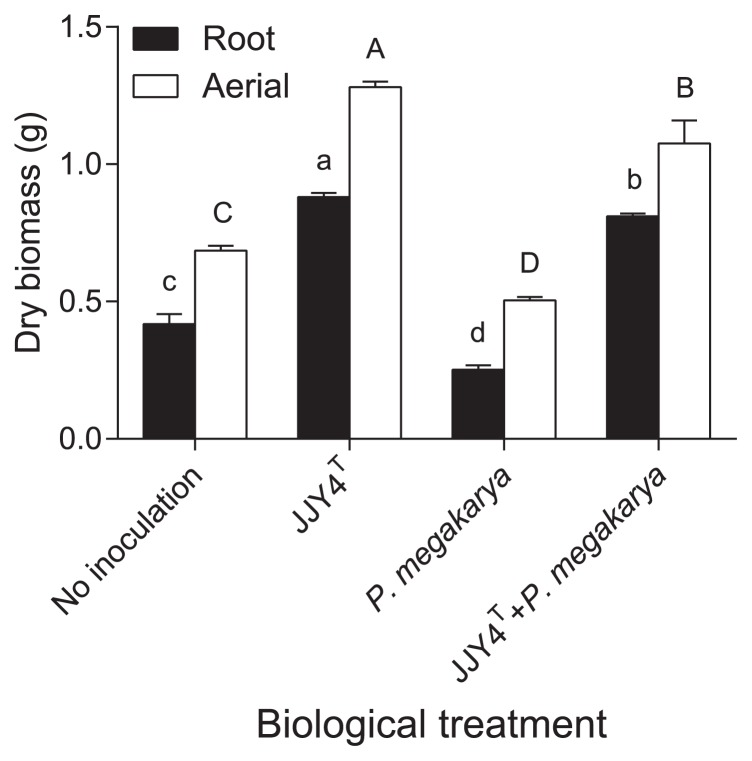
Root and aerial biomasses of 3-month-old cocoa plantlets grown in different substrates: non-inoculated, inoculated with strain JJY4^T^, inoculated with *Phytophthora megakarya*, or co-inoculated with strain JJY4^T^ and *P. megakarya*. Values with different letters are significantly different (LSD test, root biomass: lower case, aerial biomass: upper case).

JJY4^T^ produced siderophores and IAA, solubilized inorganic phosphate (14), and degraded 1-aminocyclopropane-1-carboxylate (ACC deaminase activity), an intermediary product in the biosynthetic pathway of the plant hormone ethylene (29). All these features are typically found in PGPR (8, 15) and suggest that JJY4^T^ is categorized as such. JJY4^T^ effectively showed the capacity to promote plant growth, first by accelerating cocoa seed germination. The germination rate 10 d after seeding was 92% when the growth substrate was inoculated with JJY4^T^, while it was only 42% in the control treatment. Germination rates 30 d after seeding reached 100% for both treatments. The promotion of seed germination by actinobacteria has been reported previously (13). Furthermore, cocoa growth was altered by the inoculation treatment of the substrate (*P*<0.0001). The root and aerial biomasses of cocoa plantlets were greater in the presence of JJY4^T^ than in plants grown in the sterile substrate (Fig. 1). Similar to several other actinobacteria isolated from the rhizosphere or plant tissues, JJY4^T^ exhibited the ability to promote plant growth (15, 26).

JJY4^T^ exhibited antimicrobial activities against a broad range of microorganisms. It inhibited the growth of all fungi, oomycetes, and bacteria tested in this study. This property relied at least partly on the production of geldanamycin because HPLC, mass spectrometry, and crystallography experiments led to the identification of this antibiotic in the JJY4^T^ supernatant. Geldanamycin is a type-I polyketide compound that was initially isolated from *Streptomyces hygroscopicus* var. *geldanus* (7). The biological properties of geldanamycin include antibacterial and antifungal activities (6). Geldanamycin is also known to inhibit the growth of oomycetes (7) such as *P. megakarya*, and several geldanamycin-producing strains have been identified as effective biocontrol agents of plant diseases including *Phytophthora*-induced root rot (40). The ability to synthesize geldanamycin appeared to be an important asset for some biocontrol agents since the loss of this property correlated in *S. melanosporofaciens* with an inability to protect potato tubers against common scab (1).

The geldanamycin producer JJY4^T^ protected cocoa seed and cocoa plantlets against *P. megakarya* infection. The germination rates of cocoa seeds in soils inoculated solely with *P. megakarya* or co-inoculated with the pathogen and JJY4^T^ 10 d after seeding were 83% and 17%, respectively. The germination rate 30 d after seeding reached 100% for the treatment combining JJY4^T^ and the pathogen, but was only 67% for the *P. megakarya* only treatment. Although *P. megakarya* significantly decreased plant growth more than that in plants from non-inoculated pots, the presence of JJY4^T^ protected plants from the negative effects of the pathogenic agent, while maintaining growth stimulation (Fig. 1). Although *P. megakarya* is known for its ability to cause symptoms on cocoa pods, cocoa plants may also be infected at every developmental stage (2). The pathogen has even been isolated from the asymptomatic roots of various plants growing in a cocoa plantation (2). Our results suggest that the infection of cocoa root plantlets by *P. megakarya* negatively affects seed germination and seedling development.

The foliar disc assay is commonly used to assess the sensitivity of cocoa plants to pod rot (27). In the present study, the necrosis index in foliar discs for plants grown in the substrate inoculated with JJY4^T^ was significantly lower (*P*=0.0002, *t*-test) than that from leaves of the control treatment (1.33±0.14 and 2.84±0.13, respectively). The observed systemic protective effect of this soil bacterium on cocoa plantlets suggests that JJY4^T^ stimulates systemic resistance in plants similar to other soil streptomycetes (24). We cannot exclude the direct effects of JJY4^T^ on *P. megakarya* because geldanamycin-producing actinobacterial isolates have been found to live as endophytes within cocoa (26).

**Fig. 2:**
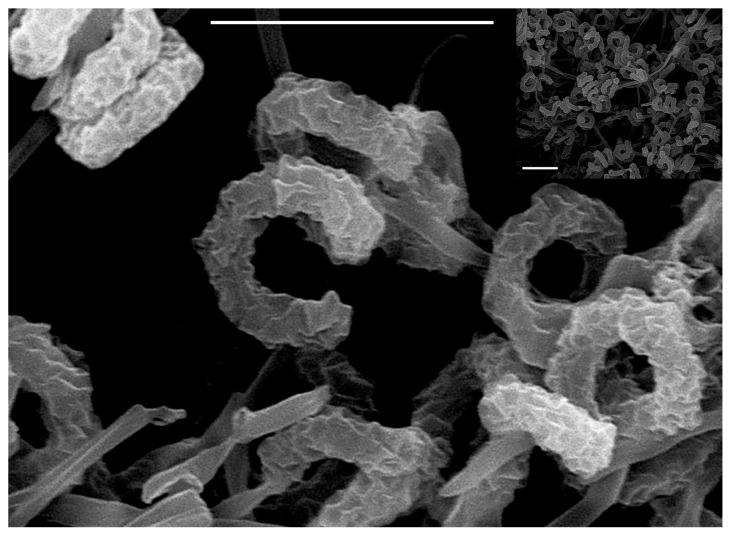
Scanning electron micrograph showing the formation of spiral chains of rugose-ornamented spores by strain JJY4^T^ grown on yeast extract-malt extract (ISP-2 medium) agar at 30°C for 14 d. Bars represent 5 μm.

The ability to produce geldanamycin is a common characteristic of members of the *S. violaceusniger* clade (12). In order to clarify whether strain JJY4^T^ also belongs to this clade, various characterization tests were performed. Strain JJY4^T^ showed the typical morphology within the genus *Streptomyces* (Table 1). It exhibited good growth on ISP-2, oat-meal (ISP-3), ISP-4, GB, and modified Bennett’s agars. The strain formed a yellow substrate mycelium and a grey and abundant aerial spore mass on ISP-2 agar. It produced brownish soluble pigments on ISP-2 agar, but no diffusible pigment on ISP-3 agar. The aerial hyphae of strain JJY4^T^ differentiated into long, spiral chains of cylindrical spores with rugose ornamentation (Fig. 2). All these characteristics strongly suggest that isolate JJY4^T^ is a member of the *S. violaceusniger* clade (12, 23, 34).

The cell wall of JJY4^T^ contained ll-2,6-diaminopimelic acid. The predominant menaquinones were MK9-(H_6_) and MK9-(H_4_) (57% and 21%, respectively). Minor menaquinones were MK9-(H_8_) (8%) and MK9-(H_2_) (4%). Polar lipids were phosphatidylglycerol, phosphatidylinositol, phosphatidylethanolamine, hydroxy-phosphatidylethanolamine, aminolipid, unidentified phospholipids, and an unidentified polar lipid (Fig. S1). The cellular fatty acid composition of JJY4^T^ is typical of the genus *Streptomyces*. The major fatty acids were iso-C_16:0_ (22.5%), anteiso-C_15:0_ (17.6%), anteiso-C_17:0_ (13.6%) and C_16:0_ (10.2%), and iso-C_15:0_ (9.7%) (see Table S1 for the complete composition).

**Fig. 3:**
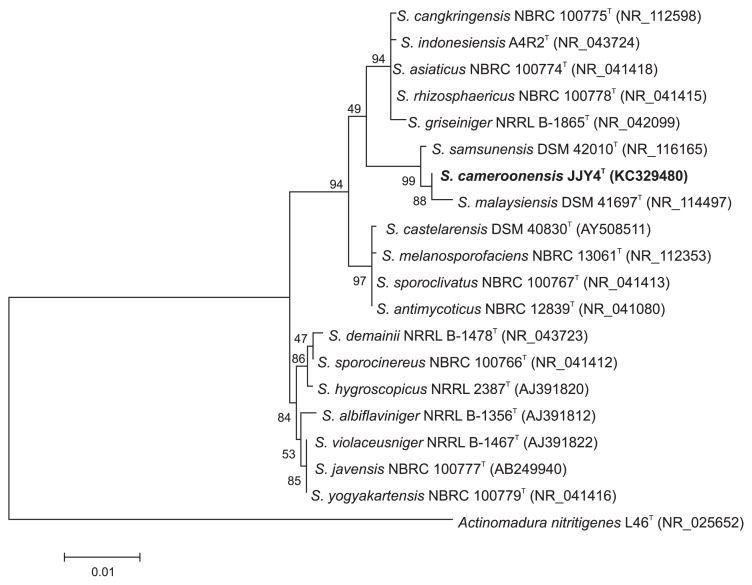
Maximum likelihood phylogenetic tree, based on nearly complete 16S rRNA gene sequences (1473 nt) showing the position of strain JJY4^T^ on the *Streptomyces violaceusniger* gene tree. The bar represents a distance of 0.01 substitutions per nucleotide.

The 16S rRNA gene sequencing of JJY4^T^ confirmed that this strain belongs to the genus *Streptomyces*. Comparisons with the type strain sequences of other *Streptomyces* species classified strain JJY4^T^ in the *S. violaceusniger* clade, which formed a subclade with *Streptomyces malaysiensis* DSM 41697^T^ and *Streptomyces samsunensis* DSM 42010^T^ (Fig. 3 and Fig. S2). Strain JJY4^T^ shared a 16S rRNA gene sequence similarity of 99.8% with *S. samsunensis* DSM 42010^T^ (3 nt differences at 1455 locations) and 99.6% with *S. malaysiensis* DSM 41697^T^ (6 nt differences at 1456 locations). Other type strains used to construct phylogenetic trees shared a 16S rRNA gene sequence similarity of less than 99.0%. However, strain JJY4^T^ may be distinguished from the two latter type strains according to the method estimating DNA–DNA relatedness from whole-genome DNA. The Δ*T_m_* values between hybrid DNA duplexes and homologous DNA were 5.6°C and 7.6°C for *S. malaysiensis* DSM 41697^T^ and *S. samsunensis* DSM 42010^T^, respectively. This is above the recommended Δ*T_m_* value (5°C) to distinguish bacterial species (41), and, thus, JJY4^T^ may be regarded as a novel species.

The phenotypic properties of strain JJY4^T^, and its most closely related *Streptomyces* type strains (*i.e. S. samsunensis* and *S. malaysiensis*), are shown in Table 2. Several studies have reported the use of physiological and biochemical characterization to identify *Streptomyces* species (1, 19, 32). Strain JJY4^T^ was found to have the ability to hydrolyze arbutin, hypoxanthine, l-tyrosine, and Tween 80, but not aesculin or gelatin (Table 2). It also utilized all carbon and nitrogen sources tested in the present study, with the exception of cellobiose and l-isoleucine (Table 2). Antibiotic susceptibility testing showed that JJY4^T^ was resistant to chloramphenicol, penicillin, cephaloridine, lincomycin, rifampicin, oleandomycin, and geldanamycin, but was susceptible to kanamycin, streptomycin, and vancomycin. The growth pH range of strain JJY4^T^ was between pH 5.0 and pH 10.0. Collectively, the phenotypic properties of strain JJY4^T^ clearly differ from those of the two *Streptomyces* type strains used here for comparison. The similarity index (42) between strain JJY4^T^ and its closest relatives, *S. samsunensis* DSM 42010^T^ (32), *S. malaysiensis* DSM 41697^T^ (3), *S. indonesiensis* A4R2^T^ (34), and *S. griseiniger* NRRL B-1865^T^ (12) varied between 52 and 62%. Although strain JJY4^T^ is closely related to *S. malaysiensis* DSM 41697^T^ and *S. samsunensis* DSM 42010^T^ based on its 16S rRNA gene sequence, this strain is undoubtedly representative of a novel species of the *S. violaceusniger* clade in view of the genotypic, morphological, and phenotypic data presented here. Therefore, this novel species is proposed as *Streptomyces cameroonensis* sp. nov.

### Description of *Streptomyces cameroonensis* sp. nov

*Streptomyces cameroonensis* (cam.er.oon.en’sis. N.L. masc. adj. cameroonensis belonging/pertaining to Cameroon, the source of the organism).

Gram-positive, aerobic actinobacterium that forms extensively branched substrates and aerial hyphae that differentiate into tight, spiral spore chains. The spore surface is rugose. Grows well on ISP-2, ISP-3, ISP-4, GB, and modified Bennett’s agars. On ISP-2 medium, the aerial spore mass color is white at 4 d, becoming grey and moist when mature; the substrate mycelium is brownish-yellow and diffusible pigments are produced on ISP-2 and ISP-4 agars. Melanin pigments are not produced on ISP-7 agar. Growth occurs between 25°C and 37°C, but not at 4°C or 45°C, and from pH 5.0 to pH 9.0, but not at pH 4.0. Degrades arbutin, casein, starch, cellulose, pectin, chitin, chitosan, hypoxanthine, l-tyrosine, and Tween 80, but not aesculin or gelatin. Utilizes adonitol, dextrin, raffinose, salicin, *myo*-inositol, sodium propionate, xylose, lactose, and (+)-l-arabinose as sole carbon sources, but not cellobiose. Utilizes l-valine, l-methionine, l-phenylalanine, and l-serine as sole nitrogen sources, but not l-isoleucine. Resistant to penicillin, chloramphenicol, cephaloridine, lincomycin, rifampicin, oleandomycin, and geldanamycin, but susceptible to kanamycin, streptomycin, and vancomycin. Exhibits antimicrobial activity against *Aspergillus niger* UdS-203, *Botrytis cinerea* B191, *Fusarium oxysporum* UdS-212, *Phytophthora erythroseptica* UdS-305, *Phytophthora infestans* UdS-302, *P. megakarya* Ngo13, *Pythium aphanidermatum* UdS-321, and *Pythium myriotylum* P60, and antibacterial activity against *Agrobacterium tumefaciens* CB-1 and *Streptomyces scabiei* EF-35. Shows ACC deaminase and phosphate-solubilizing activities, produces siderophores, indole-3-acetic acid, and geldanamycin. Principal polar lipids are phosphatidylglycerol, phosphatidylinositol, phosphatidylethanolamine, and hydroxy-phosphatidylethanolamine. Predominant menaquinones are MK9-(H_6_) and MK9-(H_4_). Major cellular fatty acids are iso-C_16:0_, anteiso-C_15:0_, anteiso-C_17:0_, C_16:0_, and iso-C_15:0_. The DNA G+C content of the type strain is 73.07 mol%.

The type strain, JJY4^T^ (=NRRL B-65369, =NBRC 112705), was isolated from the rhizosphere of *Chromolaena odorata* grown in an uncropped field in Yaoundé (Cameroon). The species description is based on a single strain and, hence, serves as the type strain description.

## Supplementary material



## Figures and Tables

**Table 1:** Growth and cultural characteristics of strain JJY4^T^ and type strains of the two most closely related species of the *Streptomyces violaceusniger* clade.

Medium (with agar)	1	2	3
Yeast extract-malt extract (ISP-2)			
Growth	+++	+++	+++
Aerial mycelium	White-grey	Grey	Dark grey
Reverse color	Brown-grey	Yellow-brown	Brown-grey
Soluble pigment	Brown	None	Brown
Oat-meal (ISP-3)			
Growth	+++	+++	+++
Aerial mycelium	Grey	Grey	Smoky black
Reverse color	Yellow	Greyish-yellow	Yellow-brown
Soluble pigment	None	None	None
Starch-inorganic salts (ISP-4)			
Growth	+++	+++	+++
Aerial mycelium	Grey-green	Grey	Smoky black
Reverse color	Yellowish	Grey-green	Gris
Soluble pigment	Yellow	None	Yellow
Glycerol asparagine (ISP-5)			
Growth	++	++	+
Aerial mycelium	Grey	Grey	Grey-white
Reverse color	Light yellow	Brown	Pale yellow-grey
Soluble pigment	None	None	None
Tyrosine (ISP-7)			
Growth	++	+++	+++
Aerial mycelium	Grey	Grey	Grey
Reverse color	Brown	Brown	Brown
Soluble pigment	None	None	Dark brown
Modified Bennett’s			
Growth	+++	+++	++
Aerial mycelium	Brown	Grey	White
Reverse color	Yellow	Yellow-green	Brown
Soluble pigment	None	None	Light-brown
Glycerol bouillon (GB)			
Growth	+++	+++	+++
Aerial mycelium	Grey	Grey	Dark grey
Reverse color	Yellow	Brown	Brown grey
Soluble pigment	None	None	Brown

1, JJY4^T^; 2, *S. samsunensis* DSM 42010^T^ (32); 3, *S. malaysiensis* DSM 41697^T^ (3).

+, poor growth; ++, moderate growth; +++, good growth.

**Table 2:** Phenotypic characteristics that distinguish strain JJY4^T^ from the two most closely related species of the genus *Streptomyces*.

Characteristic	1	2	3
Degradation tests (% [w/v])			
Aesculin hydrolysis (0.1)	−	−	+
Arbutin hydrolysis (0.1)	+	+	+
Gelatin (0.4)	−	+	+
Hypoxanthine (0.4)	+	−	+
l-Tyrosine	+	+	+
Tween 80 (1.0)	+	+	+
Growth on sole carbon sources (% [w/v])			
Adonitol (1.0)	+	+	+
(+)-l-Arabinose (1.0)	+	+	+
Cellobiose (1.0)	−	+	+
Dextrin (1.0)	+	+	+
Lactose (1.0)	+	+	+
Salicin (1.0)	+	−	+
* myo-*Inositol (1.0)	+	−	+
Raffinose (1.0)	+	+	+
Xylose (1.0)	+	−	+
Sodium propionate (0.1)	+	−	−
Growth on sole nitrogen sources (0.1% [w/v])			
l-Isoleucine	−	+	+
l-Methionine	−	+	−
l-Serine	+	+	+
l-Phenylalanine	+	+	+
l-Valine	+	+	+
Growth at pH 4.0	−	−	+
Growth with 8 μg chloramphenicol mL^−1^	+	+	−

Strains were grown and tested simultaneously under the same conditions. Taxa are as in Table 1.

+, utilized; −, negative or not utilized.
