# Supplementary material for: Streptomyces cameroonensis sp. nov., a Geldanamycin Producer That Promotes Theobroma cacao Growth
Source: Microbes Environ. 2017 Mar 4;32(1):24–31. doi: 10.1264/jsme2.ME16095 (PMC5371071; doi:10.1264/jsme2.ME16095)
Supplement: Supplementary file 1 [file 32_24_s1.pdf]

Supplementary material for article

Streptomyces cameroonensis sp. nov., a geldanamycin producer that promotes *Theobroma cacao* growth

BY

THADDÉE BOUDJEKO[1,2,3]*, ROMARIC ARMEL MOUAFO TCHINDA[1,3], MINA ZITOUNI[3], JOËLLE AIMÉE VERA TCHATCHOU NANA[1], SYLVAIN LERAT[3] and CAROLE BEAULIEU[3]

[1]*Laboratory of Phytoprotection and Valorization of Plants Resources, Biotechnology Centre - Nkolbisson; P.O. BOX 3851, Messa, Yaoundé, Cameroon*

[2]*Department of Biochemistry, Faculty of Science, University of Yaoundé I, P.O. Box 812 Yaoundé, Cameroon*

[3]*Centre SÈVE, Département de Biologie, Université de Sherbrooke, Sherbrooke, Quebec J1K 2R1, Canada*

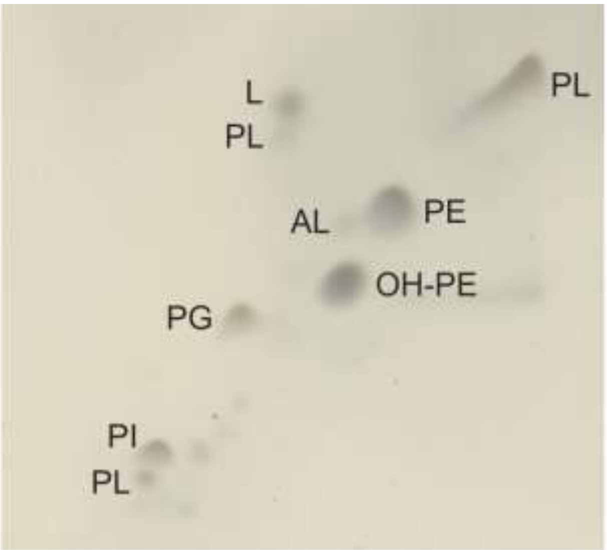

L: lipid
AL: aminolipid
PL: phospholipid
PG: phosphatidylglycerol
PI: phosphatidylinositol
PE: phosphatidylethanolamine
OH-PE: hydroxy-phosphatidylethanolamine

Supplementary Figure S1. Polar lipids produced by strain JJY4^{T} grown in ISP-2 medium.

Supplementary Table S1. Cellular fatty acids composition of strain JJY4^{T}.

Fatty acid name	Percent (%)
13:0 iso	0.30
14:0 iso	2.83
14:0	0.71
15:0 iso	9.68
15:0 anteiso	17.55
15:1 ω6*c*	0.41
15:0	1.83
16:1 iso H	1.48
16:0 iso	22.54
16:1 ω7*c* /15:0 iso 2OH	4.89
16:0	10.23
iso 17:1 ω9*c*	2.69
anteiso 17:1 ω9*c*	1.96
17:0 iso	5.20
17:0 anteiso	13.13
17:1 ω8*c*	0.67
17:0 cyclo	2.02
18:0	1.13
18:0 iso	0.47
18:1 ω7*c*	0.27

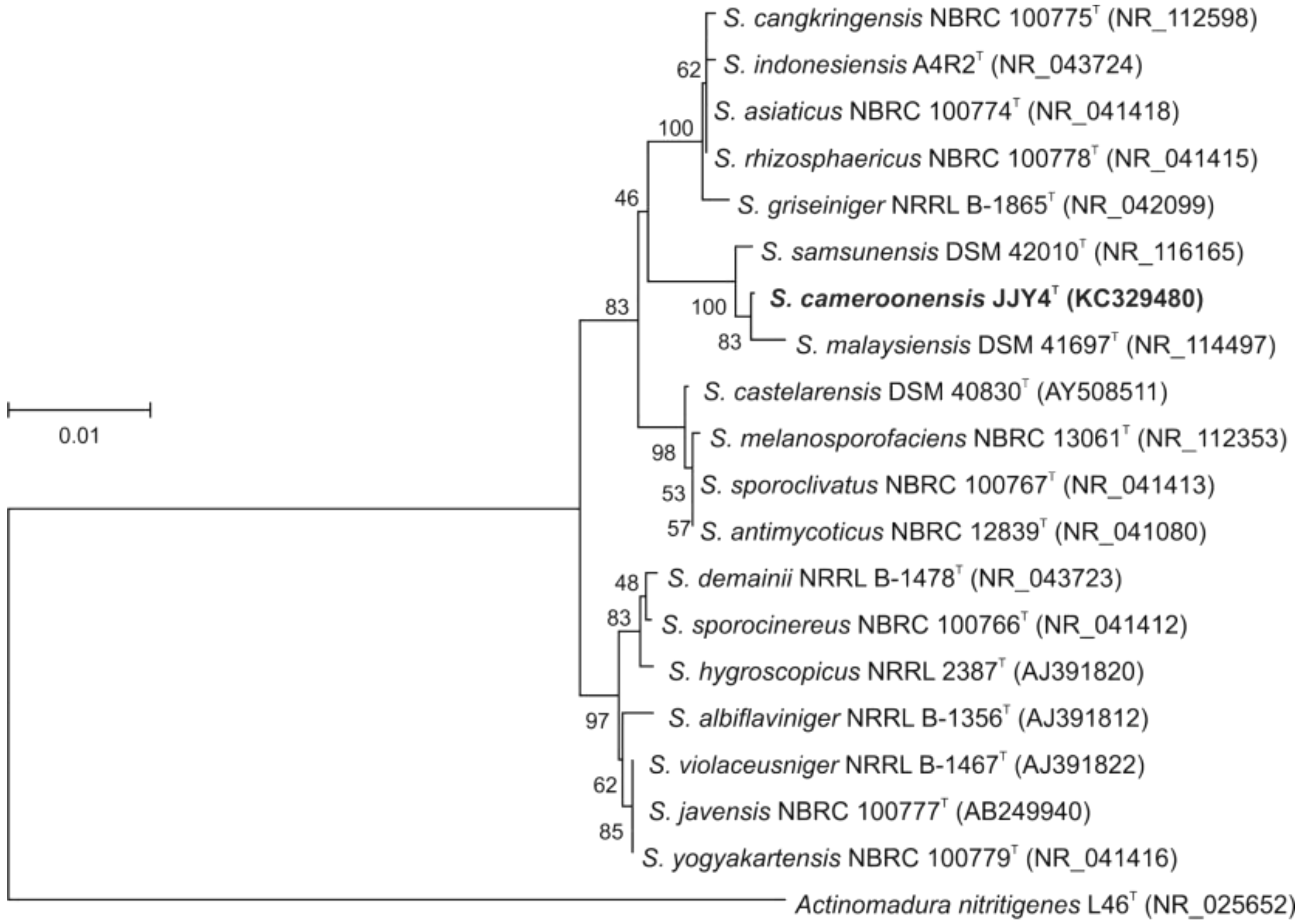

Supplementary Figure S2. Position of strain JJY4^{T} on the *S. violaceusniger* gene tree determined by the neighbor-joining method. The bar represents a distance of 0.01 substitutions per nucleotide.
